# Cascaded Multi-view Canonical Correlation (CaMCCo) for Early Diagnosis of Alzheimer’s Disease via Fusion of Clinical, Imaging and Omic Features

**DOI:** 10.1038/s41598-017-03925-0

**Published:** 2017-08-15

**Authors:** Asha Singanamalli, Haibo Wang, Anant Madabhushi, Alzheimer’s Disease Neuroimaging Initiative, Michael Weiner, Paul Aisen, Ronald Petersen, Clifford Jack, William Jagust, John Trojanowki, Arthur Toga, Laurel Beckett, Robert Green, Andrew Saykin, John Morris, Leslie Shaw, Jeffrey Kaye, Joseph Quinn, Lisa Silbert, Betty Lind, Raina Carter, Sara Dolen, Lon Schneider, Sonia Pawluczyk, Mauricio Beccera, Liberty Teodoro, Bryan Spann, James Brewer, Helen Vanderswag, Adam Fleisher, Judith Heidebrink, Joanne Lord, Sara Mason, Colleen Albers, David Knopman, Kris Johnson, Rachelle Doody, Javier Villanueva-Meyer, Munir Chowdhury, Susan Rountree, Mimi Dang, Yaakov Stern, Lawrence Honig, Karen Bell, Beau Ances, John Morris, Maria Carroll, Mary Creech, Erin Franklin, Mark Mintun, Stacy Schneider, Angela Oliver, Daniel Marson, Randall Griffith, David Clark, David Geldmacher, John Brockington, Erik Roberson, Marissa Natelson Love, Hillel Grossman, Effie Mitsis, Raj Shah, Leyla deToledo-Morrell, Ranjan Duara, Daniel Varon, Maria Greig, Peggy Roberts, Marilyn Albert, Chiadi Onyike, Daniel D’Agostino, Stephanie Kielb, James Galvin, Brittany Cerbone, Christina Michel, Dana Pogorelec, Henry Rusinek, Mony de Leon, Lidia Glodzik, Susan De Santi, P. Doraiswamy, Jeffrey Petrella, Salvador Borges-Neto, Terence Wong, Edward Coleman, Charles Smith, Greg Jicha, Peter Hardy, Partha Sinha, Elizabeth Oates, Gary Conrad, Anton Porsteinsson, Bonnie Goldstein, Kim Martin, Kelly Makino, M. Ismail, Connie Brand, Ruth Mulnard, Gaby Thai, Catherine Mc-Adams-Ortiz, Kyle Womack, Dana Mathews, Mary Quiceno, Allan Levey, James Lah, Janet Cellar, Jeffrey Burns, Russell Swerdlow, William Brooks, Liana Apostolova, Kathleen Tingus, Ellen Woo, Daniel Silverman, Po Lu, George Bartzokis, Neill Graff-Radford, Francine Parfitt, Tracy Kendall, Heather Johnson, Martin Farlow, Ann Marie Hake, Brandy Matthews, Jared Brosch, Scott Herring, Cynthia Hunt, Christopher Dyck, Richard Carson, Martha MacAvoy, Pradeep Varma, Howard Chertkow, Howard Bergman, Chris Hosein, Sandra Black, Bojana Stefanovic, Curtis Caldwell, Ging-Yuek Robin Hsiung, Howard Feldman, Benita Mudge, Michele Assaly, Elizabeth Finger, Stephen Pasternack, Irina Rachisky, Dick Trost, Andrew Kertesz, Charles Bernick, Donna Munic, Marek-Marsel Mesulam, Kristine Lipowski, Sandra Weintraub, Borna Bonakdarpour, Diana Kerwin, Chuang-Kuo Wu, Nancy Johnson, Carl Sadowsky, Teresa Villena, Raymond Scott Turner, Kathleen Johnson, Brigid Reynolds, Reisa Sperling, Keith Johnson, Gad Marshall, Jerome Yesavage, Joy Taylor, Barton Lane, Allyson Rosen, Jared Tinklenberg, Marwan Sabbagh, Christine Belden, Sandra Jacobson, Sherye Sirrel, Neil Kowall, Ronald Killiany, Andrew Budson, Alexander Norbash, Patricia Lynn Johnson, Thomas Obisesan, Saba Wolday, Joanne Allard, Alan Lerner, Paula Ogrocki, Curtis Tatsuoka, Parianne Fatica, Evan Fletcher, Pauline Maillard, John Olichney, Charles DeCarli, Owen Carmichael, Smita Kittur, Michael Borrie, T-Y Lee, RobBartha, Sterling Johnson, Sanjay Asthana, Cynthia Carlsson, Steven Potkin, Adrian Preda, Dana Nguyen, Pierre Tariot, Anna Burke, Nadira Trncic, Adam Fleisher, Stephanie Reeder, Vernice Bates, Horacio Capote, Michelle Rainka, Douglas Scharre, Maria Kataki, Anahita Adeli, Earl Zimmerman, Dzintra Celmins, Alice Brown, Godfrey Pearlson, Karen Blank, Karen Anderson, Laura Flashman, Marc Seltzer, Mary Hynes, Robert Santulli, Kaycee Sink, Leslie Gordineer, Jeff Williamson, Pradeep Garg, Franklin Watkins, Brian Ott, Henry Querfurth, Geoffrey Tremont, Stephen Salloway, Paul Malloy, Stephen Correia, Howard Rosen, Bruce Miller, David Perry, Jacobo Mintzer, Kenneth Spicer, David Bachman, Nunzio Pomara, Raymundo Hernando, Antero Sarrael, Norman Relkin, Gloria Chaing, Michael Lin, Lisa Ravdin, Amanda Smith, Balebail Ashok Raj, Kristin Fargher

**Affiliations:** 2 Magnetic Resonance Unit at the VA Medical Center and Radiology, Medicine, Psychiatry and Neurology, University of California, San Francisco, USA; 3 San Diego School of Medicine, University of California, California, USA; 4 Mayo Clinic, Minnesota, USA; 5 Mayo Clinic, Rochester, USA; 6 University of California, Berkeley, USA; 7 University of Pennsylvania, Pennsylvania, USA; 8 University of Southern California, California, USA; 9 University of California, Davis, California, USA; 10 MPH Brigham and Women’s Hospital/Harvard Medical School, Massachusetts, USA; 11 Indiana University, Indiana, USA; 12 Washington University St. Louis, Missouri, USA; 13 Oregon Health and Science University, Oregon, USA; 14 University of California–San Diego, California, USA; 15 University of Michigan, Michigan, USA; 16 Baylor College of Medicine, Houston, State of Texas USA; 17 Columbia University Medical Center, South Carolina, USA; 18 University of Alabama – Birmingham, Alabama, USA; 19 Mount Sinai School of Medicine, New York, USA; 20 Rush University Medical Center, Rush University, Illinois, USA; 21 Wien Center, Florida, USA; 22 Johns Hopkins University, Maryland, USA; 23 New York University, NY, USA; 24 Duke University Medical Center, North Carolina, USA; 25 University of Kentucky, Kentucky, USA; 26 University of Rochester Medical Center, NY, USA; 27 University of California, Irvine California, USA; 28 University of Texas Southwestern Medical School, Texas, USA; 29 Emory University, Georgia, USA; 30 University of Kansas, Medical Center, Kansas, USA; 31 University of California, Los Angeles, California, USA; 32 Mayo Clinic, Jacksonville, Jacksonville, USA; 33 Yale University School of Medicine, Connecticut, USA; 34 McGill University, Montreal-Jewish General Hospital, Montreal, Canada; 35 Sunnybrook Health Sciences, Ontario, USA; 36 U.B.C. Clinic for AD & Related Disorders, Vancouver, BC, Canada; 37 Cognitive Neurology - St. Joseph’s, Ontario, USA; 38 Cleveland Clinic Lou Ruvo Center for Brain Health, Ohio, USA; 39 Northwestern University, San Francisco, USA; 40 Premiere Research Inst (Palm Beach Neurology), west Palm Beach, USA; 41 Georgetown University Medical Center, Washington DC, USA; 42 Brigham and Women’s Hospital, Massachusetts, USA; 43 Stanford University, California, USA; 44 Banner Sun Health Research Institute, Sun City, AZ 85351 USA; 45 Boston University, Massachusetts, USA; 46 Howard University, Washington DC, USA; 47 Case Western Reserve University, Ohio, USA; 48 University of California, Davis – Sacramento, California, USA; 49 Neurological Care of CNY, Liverpool, NY 13088 USA; 50 Parkwood Hospital, Pennsylvania, USA; 51 University of Wisconsin, Wisconsin, USA; 52 University of California, Irvine – BIC, USA; 53 Banner Alzheimer’s Institute, Phoenix, AZ 85006 USA; 54 Dent Neurologic Institute, NY, USA; 55 Ohio State University, Ohio, USA; 56 Albany Medical College, NY, USA; 57 Hartford Hospital, Olin Neuropsychiatry Research Center, Connecticut, USA; 58 Dartmouth-Hitchcock Medical Center, New Hampshire, USA; 59 Wake Forest University Health Sciences, North Carolina, USA; 60 Rhode Island Hospital, state of Rhode Island, Providence, RI 02903 USA; 61 Butler Hospital, Providence, Rhode Island, USA; 62 University of California, San Francisco, USA; 63 Medical University South Carolina, Charleston, SC 29425 USA; 64 Nathan Kline Institute, Orangeburg, New York USA; 65 Cornell University, Ithaca, New York USA; 66 USF Health Byrd Alzheimer’s Institute, University of South Florida, Tampa, FL 33613 USA; 1 Department of Biomedical Engineering, Case Western Reserve University, Cleveland, OH 44106 USA

## Abstract

The introduction of mild cognitive impairment (MCI) as a diagnostic category adds to the challenges of diagnosing Alzheimer’s Disease (AD). No single marker has been proven to accurately categorize patients into their respective diagnostic groups. Thus, previous studies have attempted to develop fused predictors of AD and MCI. These studies have two main limitations. Most do not simultaneously consider all diagnostic categories and provide suboptimal fused representations using the same set of modalities for prediction of all classes. In this work, we present a combined framework, cascaded multiview canonical correlation (CaMCCo), for fusion and cascaded classification that incorporates all diagnostic categories and optimizes classification by selectively combining a subset of modalities at each level of the cascade. CaMCCo is evaluated on a data cohort comprising 149 patients for whom neurophysiological, neuroimaging, proteomic and genomic data were available. Results suggest that fusion of select modalities for each classification task outperforms (mean AUC = 0.92) fusion of all modalities (mean AUC = 0.54) and individual modalities (mean AUC = 0.90, 0.53, 0.71, 0.73, 0.62, 0.68). In addition, CaMCCo outperforms all other multi-class classification methods for MCI prediction (PPV: 0.80 vs. 0.67, 0.63).

## Introduction

Alzheimer’s Disease (AD) is the most prevalent type of dementia in the US, and is primarily characterized by irreversible cognitive decline associated with neurodegeneration^1^. On account of an increasing aging population in the US, the annual incidence of AD is expected to double by 2050^2^. However, studies have shown that the number of cases in 2050 can be reduced by 50% if the average age at the onset of the disease could be delayed by 5 years^3^. This may be achieved by early diagnosis and intervention with treatments that delay disease progression.

The original diagnostic criteria, known as NINCDS-ADRDA criteria, qualitatively combined information from medical history, clinical examination, neurophysiological testing and laboratory assessments to provide a sensitivity of 81% and a specificity of 70% for AD diagnosis^4^. In an attempt to diagnose AD earlier, the revised criteria now include two major changes: (i) addition of an intermediate diagnostic group, mild cognitive impairment (MCI) as well as (ii) guidelines for interpretation of imaging and molecular markers^1^. The intermediate diagnostic category, MCI, comprises of a heterogeneous group of patients who present early symptoms of cognitive impairment which do not interrupt daily life. While some MCI patients progress to AD over time, some remain stable while a few even regress back to healthy states. Given that MCI patients are at a greater risk for AD, there is an opportunity for early diagnosis of AD by identifying the subpopulation of MCI patients who progress to AD. However, to move toward this opportunity, the most immediate challenge is to accurately distinguish MCI from both HC and AD. The new diagnostic criteria therefore includes recommendations for incorporation of alternate biomarkers that have previously shown promise in predicting presymptomatic disease^5^.

Alongside recent developments in imaging and molecular diagnostic technologies, several studies have sought to identify biomarkers of AD. Cerebrospinal fluid (CSF) markers in particular have been extensively studied^6–8^ on account of their direct relationship with pathological characteristics of the disease such as amyloid burden and neuronal degeneration. On the genetic front, apolipoprotein E (ApoE) has been established as an indicator of risk for AD^9^. Structural information on 1.5 Tesla T1w Magnetic Resonance Imaging (MRI)^10^ such as hippocampal volume and functional information on [18F] fluorodeoxyglucose uptake (FDG-PET)^11^ such as changes in glucose metabolism have previously shown to be predictive of AD. Availability of multiple, complementary markers and data streams now presents an opportunity to combine different sources of information in order to potentially improve the ability to predict AD early, prior to its onset. However, qualitatively combining the vast amount of information is challenging and likely to result in subjective interpretations. On the other hand, quantitative approaches to identification of fused biomarkers is challenged by differences in data dimensionality, small sample size of most biomedical datasets and by the increase in data dimensionality associated with combining multiscale data^12–15^.

Several methods have previously been developed and explored to quantitatively combine multiscale biomedical data. Most data fusion approaches can generally be categorized based on the level at which information is combined: (i) raw data level (low level fusion), (ii) feature level (intermediate level fusion) or (iii) decision level (high level fusion)^16^. Data integration at the raw data level is limited to homogeneous data sources and is thus not directly applicable for fusion of multiscale, biomedical data. Alternatively, decision level strategies^17^ bypass challenges associated with fusion of heterogeneous data types by combining independently derived decisions from each data source. In doing so, relationships between the different data channels remain largely unexploited^14, 15^.

Most previous work in prediction of AD employ feature level integration where raw data is first converted into quantitative feature representations which are then combined using concatenation-based^18, 19^, kernel-based^20, 21^, manifold-based^22^ and most recently deep learning-based^23, 24^ methods. A brief summary of select related previous work is provided in Table 1. While feature concatenation^18, 19^ provides a simple method for investigating the added predictive value of each modality, it is sub-optimal for combining modalities with significantly different dimensionalities as modalities with larger feature sets are likely to dominate the joint-representation and hence the fused predictor^12^. Kernel-based and manifold-based methods^20–22^ alternatively transform raw data from the original space to a high dimensional embedding space where the different data types are more homogeneously represented, thereby making them more amenable for fusion. However, such methods are prone to overfitting^25, 26^ particularly given the small sample sizes of most biomedical datasets and the noise associated with each of the biomedical data sources which, if unaccounted for, may drown the increase in signal achievable by fusion. Suk *et al*.^23^ and Liu *et al*.^24^ presented deep learning based fusion approaches which seek to learn integrated structural and functional feature representations from MRI and PET. However, the method is limited to fusion of spatially aligned imaging data. In addition, deep learning methods generally require very large datasets in order to model complex non-linear relationships via several hidden layers. This could very easily result in overfitting on datasets with small sample size, especially in the presence of noise.

**Table 1:** Summary of related previous work.

Previous Work	Modalities	Methods	N	Classes and Performance
Gray *et al*. (NeuroImage, 2013)^22^	Baseline T1w MRI, FDG PET, CSF	Joint embedding of manifolds constructed using random forest based similarity measure	147	AD/HC (Acc: 89% +/−0.7), MCI/HC (Acc: 74.6% +/−0.8), pMCI/sMCI (Acc: 58% +/−0.9)
Zhang *et al*. (NeuroImage, 2011)^20^	Baseline T1w MRI, FDG PET, CSF	Kernel combination method embedded with support vector machine classifier	202	AD/HC (Acc: 93.2%, Sen: 93%, Spec: 93.3%), MCI/HC (Acc: 76.4%, Sen: 81.8%, Spec: 66%; 91.5% pMCI and 73.4% sMCI classified as MCI)
Hinrichs *et al*. (NeuroImage, 2011)^21^	Baseline and longitudinal T1w MRI, FDG PET, cognitive measures; Baseline CSF, ApoE	Multi-kernel learning framework with support vector machine classifier	233	AD/HC (Acc: 92.4%, Sen: 86.7%, Spec: 96.6%, AUC: 0.977), pMCI/rMCI (AUC: 0.97), pMCI/sMCI (AUC: 0.77)
Westman *et al*. (NeuroImage, 2012)^39^	Baseline T1w MRI, CSF	Orthogonal partial least squares (OPLS)	369	AD/HC (Acc: 91.8%, Sen: 88.5%, Spec: 94.6%, AUC: 0.958), MCI/HC (Acc: 77.6%, Sen: 72.8%, Spec: 84.7%, AUC: 0.876), pMCI/sMCI using AD/HC model (Acc: 58.6%, 65.8%, 66.4%, 66.1%, AUC: 0.594, 0.647, 0.610, 0.578 for conversion within 12, 18, 24 and 36 months, respectively)
Da *et al*. (NeuroImage, 2014)^18^	Baseline T1w MRI, Cognitive scores, CSF, ApoE	SVM Classification of concatenated features	432, 381	AD/HC (T1w MRI AUC: 0.98), sMCI/pMCI Kalpan Meier analysis
Davatzikos *et al*. (Neurobiology, 2011)^19^	Baseline T1w MRI (SPARE-AD), CSF	SVM Classification of concatenated features; pMCI and sMCI categorization based on global CDR score change at follow-up (6–36 months)	239	sMCI/pMCI (T1w MRI AUC: 0.734, T1w MRI + CSF AUC: 0.671)
Suk *et al*. (NeuroImage, 2014)^23^	Baseline T1w MRI, FDG PET	Joint feature representation of image patches using Deep Boltzman Machine (DBM)	194, 305, 204	AD/HC (Acc: 95.35%, AUC: 0.9877), MCI/HC (Acc: 85.67%, AUC: 0.88), pMCI/sMCI (Acc: 75.92%, AUC: 0.747)
Zhu *et al*. (NeuroImage, 2014)^40^	T1w MRI, FDG PET, CSF	Feature selection method and regression to predict clinical variables in addition to class labels	202	AD/HC (Acc: 95.9%, AUC: 98.8), MCI/HC (Acc: 82.0%, AUC: 87.0), sMCI/pMCI (Acc: 72.6%, AUC: 78.8%)
Liu *et al*. (IEEE TMI, 2015)^24^	Baseline T1w MRI, FDG PET	Fused data representation of image patches using stacked autoencoder for multiclass classification	331	AD/HC (Multiclass Precision: 59.1 +/− 19.7, 52.2 +/− 11.8, 40.2 +/− 14.4, 64.1 +/− 15.24 for HC, sMCI, pMCI and AD, Acc: 53.8 +/− 4.8, Sen: 52.1 +/− 11.8, Spe:87 +/− 9.6)

Regardless of the fusion strategy employed, most previous studies evaluate their methods by simplifying the multiclass problem (HC vs. MCI vs. AD) into the following binary classification tasks – AD vs. HC, MCI vs. HC. Recent work^24^ showed that multiclass classification resulted in significantly lower predictive performance as compared to that of the aforementioned binary classification tasks, suggesting that all the diagnostic classes must be considered to estimate the performance of a proposed model in a clinical setting. Generally, there are three common methods for multiclass classification – one vs. another, one vs. all (OVA) and one shot classification (OSC). For classification task with ${\mathscr{C}}=\{{c}_{1},{c}_{2},\ldots {c}_{n}\}$ classes, the one vs. another classifier attempts to independently solve binary class problems *c*_*i*_ vs. *c*_*j*_, $i\ne j$ arising from all pairwise combinations of classes. With this strategy, it is unclear how to combine results from the multiple binary problems in order to then determine the overall classifier performance. Alternatively, OVA seeks to solve *c*_*i*_ vs {*c*_*j*_}, *j* = 1 … *n*, $j\ne i$, while OSC attempts to simultaneously solve *c*_1_ vs. *c*_2_ vs. … vs. *c*_*n*_. OVA may not be able to appropriately classify intermediate classes such as MCI where the ‘all’ category comprises of data points that lie on either extrema of disease spectrum (i.e. healthy and AD). While OSC, which classifies multiple classes at once, overcomes the aforementioned limitations of the other two strategies, it assumes that the same set of modalities are optimal for separating all classes. When addressing multiclass problem in the context of data fusion, it may not be realistic to expect the same combination of modalities to be the most informative for all the various classes. In addition, some classification tasks may require information from fewer modalities to provide sufficiently accurate information while other, more challenging tasks may require additional information.

**Figure 1:**
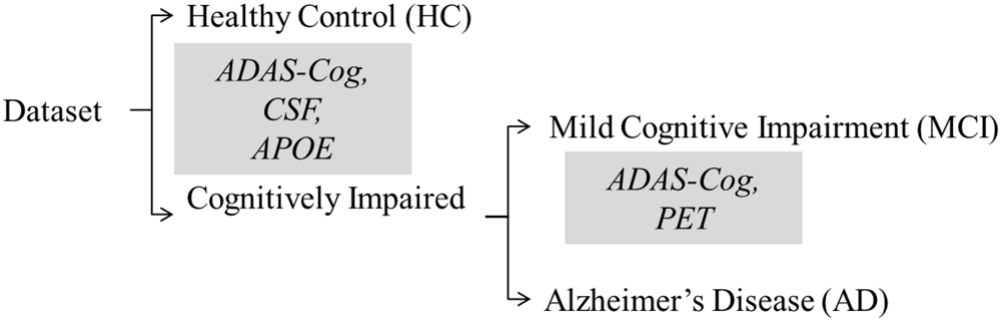
The cascade and the modalities for fusion at each level of the cascade were determined on training set and validated on independent testing set. Neurophysiological test scores (ADAS-Cog) are fused with CSF proteomics and APOE at the first level of the cascade to identify healthy controls (HC). At the second level, ADAS-Cog scores are combined with PET to distinguish between patients with Alzheimer’s Disease (AD) and mild cognitive impairment (MCI).

In this work, we introduce the cascaded multiview canonical correlation (CaMCCo) framework which brings together three different unique ideas; data fusion approach, modality selection concept and a cascaded classification scheme. The CaMCCo approach is employed in this paper for the problem of AD diagnosis. CaMCCo seeks to fuse a subset of modalities from T1w MRI, FDG PET, ApoE, CSF, plasma proteomics and neurophysiological exam scores in order to optimize classifier performance at each level of the cascade (Fig. 1). For data fusion, CaMCCo employs supervised multiview canonical correlation analysis (sMVCCA)^27, 28^ which provides a common, low dimensional representation that is discriminative of classes and allows for combining any number of heterogeneous forms of multidimensional, multimodal data. The fusion scheme operates under the assumption that information overlap increases with increasing number of data sources or ‘views’ as all views fundamentally capture information pertaining to the same object. As such, it seeks to maximize correlations between modalities and with class labels.

Previous work^27, 28^ has shown the application of sMVCCA in the context of predicting prostate cancer prognosis where sMVCCA based fusion of histologic and proteomic features was found to be more discriminative of classes as compared to individual modalities as well as several fused representations including LDA, CCA, MVCCA, PCA, regularized CCA (RCCA), supervised regularized CCA (SRCCA), and generalized embedding concatenation (GEC). Although sMVCCA is able to fuse any number of modalities, the practicality of its application in a clinical setting where the trade-off between added improvement in performance and increased burden of additional tests must be leveraged^29^. CaMCCo therefore extends on previous work to address clinical challenges associated with AD diagnosis by employing the fusion methodology within cascaded classification framework where only a subset of modality(ies) that maximize the performance for each classification task are fused at each level of cascade. Unlike most prior applications of multiclass classification methods to AD diagnosis, CaMCCo simultaneously considers all diagnostic classes via its cascaded classification approach. Unlike most prior applications of data fusion methods for AD diagnosis, CaMCCo seeks to identify, selectively retain and combine only the most informative data source(s) for each class label. As shown in Fig. 1, each patient is first classified as being healthy or cognitively impaired (CI) using ADAS-Cog score, CSF and APOE. If classified as CI, the ADAS-Cog and PET are used to distinguish between MCI and AD cases.

## Methods

### Supervised Multiview Canonical Correlation Analysis for Data Fusion

We apply supervised Multiview Canonical Correlation Analysis (sMVCCA)^27, 28^, an extension of canonical correlation analysis (CCA) and multiview canonical correlation analysis (MVCCA)^30^, to obtain a low-dimensional, shared representation of the modalities of interest. CCA^31^ is a linear dimensionality reduction method commonly used for data fusion as it accounts for relationships between two sets of input variables. MVCCA generalizes CCA by finding the linear subspace where pairwise correlations between multiple (more than two) modalities can be maximized. However, both CCA and MVCCA are unsupervised and therefore do not guarantee a subspace that is optimal for class separation. sMVCCA is a supervised form of MVCCA where class labels are embedded as one of the variable sets. Additional details and formulations for CCA and MVCCA are provided in the appendix and the theoretical framework for sMVCCA is provided below. Table 2 provides a summary of notations used in this section.

**Table 2:** Summary of Notations.

Symbol	Description
*n*, *N*	subjects, total number of subjects
*k*, *K*	modalities, total number of modalities; **x**_*k*_, *k* ∈ {1, …, *k*}
*m*, *M*_*k*_	features, total number of features in each modality; *m* ∈ {1, …, *M*_*k*_}
*M*	total number of features over all modalities; *M* = ∑_*k*_ *M*_*k*_
**x**_*k*_	data matrix containing features from modality *k* for all subjects, ${{\mathbb{R}}}^{n\times {M}_{k}}$
**X**	concatenated data matrix containing all features from all modalities $[{{\bf{x}}}_{1},\ldots ,{{\bf{x}}}_{K}]$, ${{\mathbb{R}}}^{n\times ({M}_{1}+\ldots +{M}_{K})}$
**w**_*k*_	weight vector for modality *k*, ${{\mathbb{R}}}^{{M}_{k}\times 1}$
**W**_*k*_	weight matrix for modality *k*, ${{\mathbb{R}}}^{{M}_{k}\times n}$
**w**	concatenated weight vector over all modalities ${[{{\bf{w}}}_{1}^{T},{{\bf{w}}}_{2}^{T},\ldots ,{{\bf{w}}}_{K}^{T}]}^{T}$, ${{\mathbb{R}}}^{M\times 1}$
**W**_*x*_	weight matrix for all modalities $[{{\bf{W}}}_{1},{{\bf{W}}}_{2},\ldots ,{{\bf{W}}}_{M}]$, ${{\mathbb{R}}}^{M\times n}$
**Y**	label matrix ${{\mathbb{R}}}^{n\times G}$
*g*, *G*	classes, total number of classes
**W**_*y*_	notation used in sMVCCA to denote **W** for all labels ${{\mathbb{R}}}^{g\times n}$
*i*	data vector of selected modalities $i\subseteq k$
*p*	total number of features over modalities in *i*, $p={\sum }_{i}{M}_{i}$
**X**_**i**_	concatenated data matrix containing all features from a subset of modalities [**x**_*i*_], ${{\mathbb{R}}}^{n\times p}$
*d*	dimensionality of the fused data subspace

Consider a multimodal dataset ${\bf{X}}\in \{{x}_{1},\ldots ,{x}_{k},\ldots ,{x}_{K}\}$ in ${{\mathbb{R}}}^{n\times M}$, where *n* is the number of subjects, *K* is the number of modalities and *x*_*k*_ in ${{\mathbb{R}}}^{n\times {M}_{k}}$ refers to the feature matrix of modality k containing *M*_*k*_ features. Additionally, **X** has a corresponding binary class label matrix **Y** in ${{\mathbb{R}}}^{n\times G}$, where *G* is the total number of classes. sMVCCA seeks to maximize correlation within the modalities in **X** and between **X** and **Y** as shown below

1 $$\begin{array}{ll}\mathop{\text{arg}\,\max }\limits_{{{\bf{w}}}_{1},\mathrm{...},{{\bf{w}}}_{k},\mathrm{...},{{\bf{w}}}_{K},{{\bf{w}}}_{Y}} & \sum _{k\ne 1}\sum {{\bf{w}}}_{k}^{T}{{\bf{x}}}_{k}^{T}{{\bf{x}}}_{j}{{\bf{w}}}_{j}+\sum _{k}{{\bf{w}}}_{k}^{T}{{\bf{x}}}_{k}^{T}{\bf{Y}}{{\bf{w}}}_{Y}\\ \quad \quad \quad \quad s\mathrm{.}t\mathrm{.} & {{\bf{w}}}_{1}^{T}{{\bf{x}}}_{1}^{T}{{\bf{x}}}_{1}{{\bf{w}}}_{1}=\mathrm{1,}\ldots ,{{\bf{w}}}_{K}^{T}{{\bf{x}}}_{K}^{T}{{\bf{x}}}_{K}{{\bf{w}}}_{K}=1,\,{{\bf{w}}}_{Y}^{T}{{\bf{x}}}_{Y}^{T}{{\bf{x}}}_{Y}{{\bf{w}}}_{Y}=1\end{array}$$

2 $$s\mathrm{.}t\mathrm{.}\quad {{\bf{w}}}_{1}^{T}{{\bf{x}}}_{1}^{T}{{\bf{x}}}_{1}{{\bf{w}}}_{1}=1,\ldots ,{{\bf{w}}}_{K}^{T}{{\bf{x}}}_{K}^{T}{{\bf{x}}}_{K}{{\bf{w}}}_{K}=1,{{\bf{w}}}_{Y}^{T}{{\bf{x}}}_{Y}^{T}{{\bf{x}}}_{Y}{{\bf{w}}}_{Y}=1$$

This can be expressed in a compact matrix form as follows:

$$\begin{array}{l}\mathop{{\rm{\arg }}\,{\rm{\max }}}\limits_{{{\bf{W}}}_{x},{{\bf{W}}}_{y}}\,trace({{\bf{W}}}_{x}^{T}\bar{{\bf{C}}}{{\bf{W}}}_{x})+2\times trace({{\bf{W}}}_{x}^{T}{{\bf{X}}}^{T}{\bf{Y}}{{\bf{W}}}_{y})\\ \quad \quad \quad \,\,=trace([\begin{array}{cc}{{\bf{W}}}_{x}^{T} & {{\bf{W}}}_{y}^{T}\end{array}]\,[\begin{array}{ll}\bar{{\bf{C}}} & {{\bf{X}}}^{T}{\bf{Y}}\\ {{\bf{Y}}}^{T}{\bf{X}} & {\bf{0}}\end{array}]\,[\begin{array}{c}{{\bf{W}}}_{x}\\ {{\bf{W}}}_{y}\end{array}])\\ \quad \quad \quad \,\,=trace({\hat{{\bf{W}}}}^{T}\hat{{\bf{C}}}\hat{{\bf{W}}})\\ s.t.\end{array}$$

3 $$\begin{array}{rcl}[\begin{array}{ll}{{\bf{W}}}_{x}^{T} & {{\bf{W}}}_{y}^{T}\end{array}]\,[\begin{array}{cc}{\bar{{\bf{C}}}}_{d} & {\bf{0}}\\ {\bf{0}} & {{\bf{Y}}}^{T}{\bf{Y}}\end{array}]\,[\begin{array}{c}{{\bf{W}}}_{x}\\ {{\bf{W}}}_{y}\end{array}] & = & {\bf{I}}\\ \quad \quad \quad \quad \quad \,\,\,\,\,\,\,\,\iff {\hat{{\bf{W}}}}^{T}{\hat{{\bf{C}}}}_{d}\hat{{\bf{W}}} & = & {\bf{I}}\end{array}$$

4 $${{\bf{W}}}_{1}^{T}{{\bf{C}}}_{11}{{\bf{W}}}_{1}=\cdots ={{\bf{W}}}_{K}^{T}{{\bf{C}}}_{KK}{{\bf{W}}}_{K}={{\bf{W}}}_{y}^{T}{{\bf{Y}}}^{T}{\bf{Y}}{{\bf{W}}}_{y}.$$

where **Y** is a matrix in which class labels are encoded using Soft-1-of-Class strategy^32^.

Solving Equation 3 consists of two steps: (i) Ignoring the constraint in (4) leaves us with a quadratic programming problem, whose W* corresponds to eigenvectors of the n-largest eigenvalues of a generalized eigenvalue system: ${{\bf{C}}}_{{\bf{xy}}}{\bf{W}}=\lambda {{\bf{C}}}_{{{\bf{d}}}_{{\bf{xy}}}}{\bf{W}}$; (ii) Imposing constraint (4) upon obtaining the optimal eigenvectors W* by normalizing the corresponding section of each modality: ${{\bf{W}}}_{{\bf{j}}}^{\ast \ast }={{\bf{W}}}_{{\bf{j}}}^{\ast }{({{\bf{W}}}_{{\bf{j}}}^{\ast {\bf{T}}}{{\bf{C}}}_{{\bf{jj}}}{{\bf{W}}}_{{\bf{j}}}^{\ast })}^{-\frac{1}{2}},j=1,\ldots ,k$.

### Cascaded Multi-view Canonical Correlation Analysis (CaMCCo)

**Figure 2:**
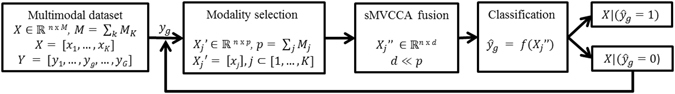
Cascaded multiview canonical correlation analysis (CaMCCo) algorithm for constructing the joint multimodal data fusion and multiclass classification framework.

As shown in Fig. 2, CaMCCo divides the classification task for a multiclass, multimodal dataset into a cascade of multiple, sequential binary classification tasks, for each of which the optimal fused representation is independently determined and provided as input to the classifier. For the multimodal dataset **X** in ${{\mathbb{R}}}^{n\times M}$, consider a label matrix **Y** in ${{\mathbb{R}}}^{n\times G}$. A subset modalities suitable for classifying class *g* from all input samples can be denoted as *i* where $i\subseteq k$. Features from modalities in *i* are concatenated to generate ${{\bf{X}}}_{{\bf{i}}}^{^{\prime} }$ in ${{\mathbb{R}}}^{n\times p}$, where $p={\sum }_{i}{M}_{i}$ and $p\le M$. The *i* modalities are fused via sMVCCA to reduce the dimensionality from *p* to *d*, where $d\ll p$, resulting in ${{\bf{X}}}_{{\bf{i}}}^{^{\prime\prime} }$. Subsequently, ${{\bf{X}}}_{{\bf{i}}}^{^{\prime\prime} }$ serves as the input to a classifier which predicts if each sample does or does not belong to class *g*, ${\hat{y}}_{g}=1$ and ${\hat{y}}_{g}=0$, respectively. The multimodal dataset consisting of only samples classified as $\hat{y}=0$ subsequently serves as the input for the next level of cascade where the modality selection, data fusion and classification steps are repeated for another class in **Y**.

Therefore, designing the cascaded classifier for CaMCCo requires determination of (a) the sequence of classification tasks that provide the best overall classifier performance, as well as the respective (b) number and (c) type of modalities to combine at each level of the cascade. In this work, these parameters were determined experimentally on the training cohort as described in Section 3.5.

## Experimental Design

### Dataset Description

Data used in the preparation of this work were obtained from the Alzheimer’s Disease Neuroimaging Initiative (ADNI) database (www.loni.ucla.edu/ADNI). The ADNI was launched in 2003 with the primary goal of testing whether serial magnetic resonance imaging (MRI), positron emission tomography (PET), other biological markers, and clinical and neuropsychological assessment can be combined to measure the progression of mild cognitive impairment (MCI) and early Alzheimer’s disease (AD). The initial goal of ADNI was to recruit 800 adults, ages 55 to 90, to participate in the research – approximately 200 cognitively normal older individuals to be followed for 3 years, 400 people with MCI to be followed for 3 years, and 200 people with early AD to be followed for 2 years (see www.adni-info.org for up-to-date information). The research protocol was approved by each local institutional review board and written informed consent. In addition to raw data, the ADNI database contains several post-processed and individually evaluated biomarkers.

In this work, we consider a subset of cases for which the following was available in the database (i) pre-computed features from T1w MRI and FDG PET, (ii) neurocognitive ADAS-cog score, (iii) complete record of CSF Proteomics, Plasma Proteomics, ApoE and (iv) clinical diagnosis at baseline. 149 ADNI participants who fulfilled the criteria were included, of which 52 were diagnosed with Alzheimer’s Disease (AD), 71 were diagnosed with mild cognitive impairment (MCI), and 26 were healthy controls (HC).

Table 3 provides the clinical and demographic details of the population considered in this study as per their diagnosis at baseline. The unique ADNI database provided RID of all patients considered in this study is provided in the Appendix.

**Table 3:** Clinical and demographic information of the 149 ADNI subjects considered in this study, selected based on the availability of imaging, non-imaging and clinical metrics at baseline.

Diagnosis	N(F/M)	Age	MMSE Score
AD	52 (16/24)	75.1 +/− 8.1	23.8 +/− 2.0
MCI	71 (17/37)	74.1 +/− 7.2	27.1 +/− 1.7
HC	26 (10/14)	74.9 +/− 7.3	28.6 +/− 1.4
Total	149 (43/75)	74.2 +/− 7.2	26.3 +/− 2.6

The dataset was split into independent training set with 60 cases (40%) and a holdout validation set with 89 cases (60%). Note that gender information was unavailable for a subset of the data, as a result of which N does not equal to the sum of females (F) and males (M).

### Feature Description

Table 4 summarizes the number and types of features considered in this study for each modality. From imaging data, we consider volumetric features extracted from T1w MRI and measures of hippocampal glucose metabolism^33, 34^ from FDG PET. Considered molecular markers include proteomic measurements from cerebrospinal fluid (CSF), plasma from the biomarker consortium and geneotype ApoE data. We additionally included a neurophysiological test score as such tests serve as the primary means for diagnosis in the current clinical setting. Although the Mini-Mental State Examination (MMSE) is the most commonly performed clinical test, we avoid using MMSE scores as they were used to determine the “ground truth” labels on which we train and test CaMCCo. Therefore, we use an alternate test score, modified Alzheimer’s Disease Assessment Scale - Cognition (ADAS-Cog) which has been used to assess the effects of experimental treatments for AD in clinical trials^35^.

**Table 4:** Summary of features considered in this study from each modality.

Modality	Features	Description	Number
Neurophysiologic Exam	Modified ADAS-Cog score^41^	Score based on cognitive test assessing memory, praxis, orientation, word recall and recognition	1
T1w MRI	Volumetric Measurements	Volumetric measures of atlas based segmented brain regions	327
FDG PET	Hippocampal Glucose Metabolism^33, 34^	Pons normalized left and right hippocampal glucose metabolism	2
CSF Proteomics	t-tau, A*β*_1–42_, p-tau_181_	Markers of neuronal degeneration, plaque formation and tau hyperphosphorylation^42^	3
Plasma Proteomics	Adiponectin, Insulin, Fibrinogen etc^43^	Concentrations of signaling proteins in blood, measured by multiplex immunoassay panel	146
ApoE Genotype	ApoE alleles 1 & 2	Combination of allele forms *ε*_2_, ${\varepsilon }_{3}$, ${\varepsilon }_{4}$	1

### Classification Model

The dataset was split into training and a holdout validation set with each comprising 40% and 60% of the data, respectively. Classification and fusion parameters were determined on the training set using 10 iterations of 5-fold stratified cross validation, upon which the optimized classifier trained on the full training set was applied to the independent validation set. Naive Bayes classifier^36^ was used to evaluate the various fused and individual modality representations. Naive Bayes is a widely used, well-established probabilistic classifier that is known to perform well on small datasets.

### Evaluation metrics

Performance measures used to evaluate each classification task include: accuracy (ACC), balanced accuracy (BACC)^37^, area under the receiver operating characteristic curve (AUC)^38^, sensitivity (SEN), specificity (SPE) and positive predictive value (PPV). The definitions and descriptions of each of these metrics are provided in Table 8 in the Appendix.

### CaMCCO Model

Class groupings and modalities selected for fusion at each level of the cascaded classification design employed by CaMCCo (Fig. 1) was determined experimentally on the training set. One-vs-all (AD vs. all, MCI vs. all, HC vs. all) classifiers were constructed and evaluated independently for each considered modality. The task that most consistently resulted in the highest AUC across all modalities served as the first level of the cascade so as to reduce error propagation. Among AD, MCI and HC, the remaining classes were assigned to the second level of the cascade.

For every classification task within the cascade, each modality was ranked based on the AUC it achieved across iterations and cross validation folds within the training set. The n highest performing modalities were fused via sMVCCA, where n was varied from 2 to 6 (total number of considered modalities). The n modalities, which in combination, provided the highest training AUC were selected.

### Comparative Strategies

CaMCCo represents a framework (${\mathcal H}$) composed of multiple modules corresponding to modality selection (${\mathscr{K}}$), multimodal data fusion (${\mathcal F}$), and multiclass classification (${\mathscr{C}}$). Accordingly, the comparative strategies against which we evaluate CaMCCo involve systematically replacing the method used for one or more of these modules with an alternative strategy. Table 5 lists the notation for each of these strategies and provides a short description.

**Table 5:** Summary of notations used to refer to comparative strategies evaluated in this work.

Symbol	Description
**Classification Methods**	
${{\mathscr{C}}}_{CAS}$	Cascaded Classifier
${{\mathscr{C}}}_{OSC}$	One Shot Classifier
${{\mathscr{C}}}_{OVA}$	One-vs-All Classifier
${{\mathscr{C}}}_{BIN}$	Binary Classifier
**Data Fusion Methods**	
${ {\mathcal F} }_{SMVCCA}$	Supervised Multiview CCA data fusion approach
${ {\mathcal F} }_{PCA}$	Principal component analysis of concatenated features as baseline fusion approach
**Modality Selection**	
${{\mathscr{K}}}_{ALL}$	Multimodal dataset comprising all modalities considered in this study
${{\mathscr{K}}}_{SEL}$	Multimodal dataset comprising select subset of all available modalities
${{\mathscr{K}}}_{MRI}$	Unimodal dataset containing quantitative attributes extracted from MRI
${{\mathscr{K}}}_{PET}$	Unimodal dataset containing quantitative attributes extracted from PET
${{\mathscr{K}}}_{CSF}$	Unimodal dataset containing proteomic measurements from CSF
${{\mathscr{K}}}_{PP}$	Unimodal dataset containing plasma proteomic data
${{\mathscr{K}}}_{APOE}$	Unimodal dataset containing APOE data
${{\mathscr{K}}}_{ADAS}$	Unimodal dataset containing ADAS-Cog scores
**Comparative Methods**	
${ {\mathcal H} }_{MRI}$ (${{\mathscr{C}}}_{CAS}$ + ${{\mathscr{K}}}_{MRI}$)	Cascaded classification of single modality MR data
${ {\mathcal H} }_{PET}$ (${{\mathscr{C}}}_{CAS}$ + ${{\mathscr{K}}}_{PET}$)	Cascaded classification of single modality PET data
${ {\mathcal H} }_{CSF}$ (${{\mathscr{C}}}_{CAS}$ + ${{\mathscr{K}}}_{CSF}$)	Cascaded classification of single modality CSF data
${ {\mathcal H} }_{PP}$ (${{\mathscr{C}}}_{CAS}$ + ${{\mathscr{K}}}_{PP}$)	Cascaded classification of single modality Plasma Proteomics data
${ {\mathcal H} }_{APOE}$ (${{\mathscr{C}}}_{CAS}$ + ${{\mathscr{K}}}_{APOE}$)	Cascaded classification of single modality APOE data
${ {\mathcal H} }_{ADAS}$ (${{\mathscr{C}}}_{CAS}$ + ${{\mathscr{K}}}_{ADAS}$)	Cascaded classification of single modality ADAS-Cog data
${ {\mathcal H} }_{CAMCCO}$ (${{\mathscr{C}}}_{CAS}$ + ${ {\mathcal F} }_{SMVCCA}$ + ${{\mathscr{K}}}_{SEL}$)	Classifier resulting from CaMCCo framework, which is comprised of cascaded classifier, sMVCCA data fusion and modality selection
${ {\mathcal H} }_{ALL}$ (${{\mathscr{C}}}_{CAS}$ + ${ {\mathcal F} }_{SMVCCA}$ + ${{\mathscr{K}}}_{ALL}$)	Cascaded classifier in combination with sMVCCA based data fusion method to combine all modalities
${ {\mathcal H} }_{PCAL}$ (${{\mathscr{C}}}_{CAS}$ + ${ {\mathcal F} }_{PCA}$ + ${{\mathscr{K}}}_{ALL}$)	Cascaded classifier with PCA reduced representation of data concatenated from all modalities
${ {\mathcal H} }_{PCA}$ (${{\mathscr{C}}}_{CAS}$ + ${ {\mathcal F} }_{PCA}$ + ${{\mathscr{K}}}_{SEL}$)	Cascaded classifier with PCA reduced representation of data concatenated from selected subset of modalities
${ {\mathcal H} }_{OVA}$ (${{\mathscr{C}}}_{OVA}$ + ${ {\mathcal F} }_{SMVCCA}$ + ${{\mathscr{K}}}_{SEL}$)	One-vs-all classifier constructed from sMVCCA fused data from selected modalities.
${ {\mathcal H} }_{OSC}$ (${{\mathscr{C}}}_{OSC}$ + ${ {\mathcal F} }_{SMVCCA}$ + ${{\mathscr{K}}}_{SEL}$)	One shot classifier constructed from sMVCCA fused data from selected modalities.
${ {\mathcal H} }_{BIN}$ (${{\mathscr{C}}}_{BIN}$ + ${ {\mathcal F} }_{SMVCCA}$ + ${{\mathscr{K}}}_{SEL}$)	Binary classifier constructed from sMVCCA fused data from selected modalities.

#### Single Modality and Multimodality Approaches

Each modality was evaluated using a single modality framework (${ {\mathcal H} }_{MRI}$, ${ {\mathcal H} }_{PET}$, ${ {\mathcal H} }_{CSF}$, ${ {\mathcal H} }_{PP}$, ${ {\mathcal H} }_{APOE}$, ${ {\mathcal H} }_{ADAS}$) consisting of cascaded classification (${{\mathscr{C}}}_{CAS}$) to ensure fair comparison with CaMCCo. In addition, we compared classification performance of CaMCCo with that of a cascaded classification model where all modalities were fused at each level of the cascade (${ {\mathcal H} }_{ALL}$).

#### Principal Component Analysis for Data Fusion

Principal Component Analysis (PCA) is a dimensionality reduction method which projects input data onto an alternate subspace defined by orthogonal basis vectors which capture the direction of variance in the data. Consider a high dimensional, concatenated multimodal data matrix ${\bf{X}}=[{x}_{1},\ldots ,{x}_{k},\ldots {x}_{K}]\in {{\mathbb{R}}}^{n\times {M}_{1}+\ldots +{M}_{k}+\ldots +{M}_{K}}$ where *K* refers to the number of modalities, *n* refers to the number of subjects, and *M*_*k*_ refers to the number of features in modality *k*.

$$[{\bf{U}},{\bf{S}},{\bf{V}}]={\rm{SVD}}(\bar{{\bf{X}}}{\bar{{\bf{X}}}}^{T})$$

Singular value decomposition is applied to mean centered data matrix, $\bar{{\bf{X}}}$, which results in **U**, **S**, **V**. The columns of ${\bf{V}}\in {{\mathbb{R}}}^{({M}_{1}+\ldots +{M}_{k}+\ldots +{M}_{K})\times ({M}_{1}+\ldots +{M}_{k}+\ldots +{M}_{K})}$ are the principal components of $\bar{{\bf{X}}}$ or the orthogonal basis vectors, ordered decreasingly by the amount of variance in the dataset explained by each component. ${\bf{U}}\in {{\mathbb{R}}}^{n\times {M}_{1}+\ldots +{M}_{k}+\ldots +{M}_{K}}$ contains the projections of $\bar{{\bf{X}}}$ on the subspace defined by **V**. ${\bf{S}}\in {{\mathbb{R}}}^{({M}_{1}+\ldots +{M}_{k}+\ldots +{M}_{K})\times ({M}_{1}+\ldots +{M}_{k}+\ldots +{M}_{K})}$ is a diagonal matrix. To reduce data dimensionality, the top *d* principal components containing most of the variance in the data are retained, onto which the data is projected.

#### Multiclass Classification

For a classification task with *G* classes, One-vs-All (OVA) method constructs *G* classifiers, each tailored to separate one class from the rest. One Shot Classification (OSC) generates a single classifier designed to simultaneously distinguish between all the classes. The last comparative strategy involves the following binary classification tasks, AD vs. HC and MCI vs. HC.

### Experiment 1: Single Modality and Multi-Modality Cascaded Classification

The objective of this experiment is to examine (i) classification performance achieved by combining multiple modalities as compared to any single modality for all classification tasks within the cascade design. In addition, the experiment seeks to determine if (ii) combining subsets of modalities tailored to optimize classification at each level of the cascade provides comparable and/or improved performance as compared to combining all the modalities for all tasks. Finally, it also evaluates (iii) the impact of the chosen fusion method on the findings for (i) and (ii).

To meet these objectives, we compare the data fusion (${\mathcal F}$) and modality selection (${\mathscr{K}}$) modules in CaMCCo (${ {\mathcal H} }_{CAMCCO}={{\mathscr{C}}}_{CAS}+{ {\mathcal F} }_{SMVCCA}+{{\mathscr{K}}}_{SEL}$) with other fusion (${ {\mathcal F} }_{PCA}$), and modality selection (${{\mathscr{K}}}_{ALL}$) approaches, including the simple single modality (${{\mathscr{K}}}_{MRI}$, ${{\mathscr{K}}}_{PET}$, ${{\mathscr{K}}}_{CSF}$, ${{\mathscr{K}}}_{PP}$, ${{\mathscr{K}}}_{APOE}$, ${{\mathscr{K}}}_{ADAS}$) classification. For individual modality experiments, PCA was applied to the experiments where the number of features were larger than the number of samples to avoid curse of dimensionality. For fused and concatenated classifiers, the number of reduced dimensions was optimized on the training set. Therefore, we consider all the combinations of modalities and fusion methods listed below:

$$\begin{array}{rcl}\,\,{ {\mathcal H} }_{ALL} & = & {{\mathscr{C}}}_{CAS}+{ {\mathcal F} }_{SMVCCA}+{{\mathscr{K}}}_{ALL}\\ \,\,{ {\mathcal H} }_{PCA} & = & {{\mathscr{C}}}_{CAS}+{ {\mathcal F} }_{PCA}+{{\mathscr{K}}}_{SEL}\\ \,{ {\mathcal H} }_{PCAL} & = & {{\mathscr{C}}}_{CAS}+{ {\mathcal F} }_{PCA}+{{\mathscr{K}}}_{ALL}\\ \,\,{ {\mathcal H} }_{MRI} & = & {{\mathscr{C}}}_{CAS}+{{\mathscr{K}}}_{MRI}\\ \,\,{ {\mathcal H} }_{PET} & = & {{\mathscr{C}}}_{CAS}+{{\mathscr{K}}}_{PET}\\ \,\,{ {\mathcal H} }_{CSF} & = & {{\mathscr{C}}}_{CAS}+{{\mathscr{K}}}_{CSF}\\ \,\,\,{ {\mathcal H} }_{PP} & = & {{\mathscr{C}}}_{CAS}+{{\mathscr{K}}}_{PP}\\ { {\mathcal H} }_{APOE} & = & {{\mathscr{C}}}_{CAS}+{{\mathscr{K}}}_{APOE}\\ { {\mathcal H} }_{ADAS} & = & {{\mathscr{C}}}_{CAS}+{{\mathscr{K}}}_{ADAS}\end{array}$$

### Experiment 2: Comparison of Multi-Class Classification Strategies for Fused Predictors

The objective of this experiment is to compare the cascaded classification method ${{\mathscr{C}}}_{CAS}$ used in CaMCCo (${ {\mathcal H} }_{CAMCCO}={{\mathscr{C}}}_{CAS}+{ {\mathcal F} }_{SMVCCA}+{{\mathscr{K}}}_{SEL}$) with other multiclass classification methods including OVA (${{\mathscr{C}}}_{OVA}$) and OSC (${{\mathscr{C}}}_{OSC}$). To ensure that only the classification module of the CaMCCo framework is evaluated, comparative classification strategies are combined with the same data fusion (${ {\mathcal F} }_{SMVCCA}$) and modality selection method (${{\mathscr{K}}}_{SEL}$) as CaMCCo. Therefore,${ {\mathcal H} }_{OVA}={{\mathscr{C}}}_{OVA}+{ {\mathcal F} }_{SMVCCA}+{{\mathscr{K}}}_{SEL}$, and ${ {\mathcal H} }_{OSC}={{\mathscr{C}}}_{OSC}+{ {\mathcal F} }_{SMVCCA}+{{\mathscr{K}}}_{SEL}$. As with CaMCCo, the optimal set of modalities to combine for each classification task associated with OVA and OSC are determined experimentally from the training set.

### Experiment 3: Evaluation of Fused Representation on Binary Classification Tasks

We perform binary classification (${{\mathscr{C}}}_{BIN}$) for the following two sets of classes, HC vs. AD and MCI vs. HC, in order to allow for direct comparison of the performance of fusion approach used in CaMCCo (${ {\mathcal F} }_{SMVCCA}$ + ${{\mathscr{K}}}_{SEL}$) with that reported in literature. As with CaMCCo, the optimal set of modalities to combine for each classification task are determined experimentally from the training set. In addition, we also report binary classification results achieved by individual modalities to examine the effect of fusion for these classification tasks and also to gain insight into the differences in classifier performance on account of the data cohort used in this study as compared to those in other studies.

## Results and Discussion

### Experiment 1: Single Modality and Multi-Modality Cascaded Classification

**Figure 3:**
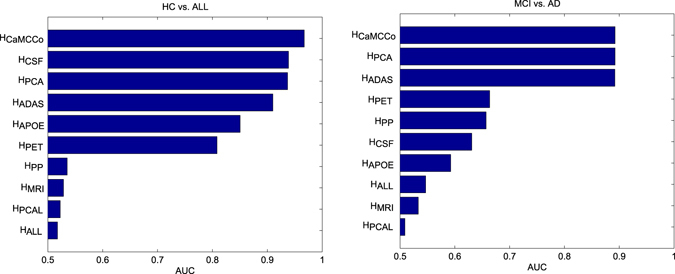
Performance of single and multi modality cascaded classifiers. Area under the ROC curve (AUC) for prediction of (**a**) healthy control (HC) from all cognitive impairments, and (**b**) mild cognitive impairment (MCI) from Alzheimer’s Disease (AD).

Figure 3 shows the performance of cascaded classifier when applied to (i) single modalities (${ {\mathcal H} }_{MRI}$, ${ {\mathcal H} }_{PET}$, ${ {\mathcal H} }_{CSF}$, ${ {\mathcal H} }_{PP}$, ${ {\mathcal H} }_{APOE}$, ${ {\mathcal H} }_{ADAS}$), (ii) fusion of all modalities (${ {\mathcal H} }_{ALL}$, ${ {\mathcal H} }_{PCAL}$) and (iii) fusion of selected modalities with multiple fusion methods (${ {\mathcal H} }_{CAMCCO}$, ${ {\mathcal H} }_{PCA}$) for prediction of HC, MCI and AD on the testing cohort. As shown in Fig. 1, ADAS-Cog, CSF and APOE were combined at the first cascade level (HC vs. All) and ADAS-Cog, and PET were combined at the second level (AD vs. MCI) in both ${ {\mathcal H} }_{CAMCCO}$ and ${ {\mathcal H} }_{PCA}$. For HC vs. all, ${ {\mathcal H} }_{CAMCCO}$ shows higher performance (AUC = 0.97) as compared to all individual modalities (max AUC = 0.93), ${ {\mathcal H} }_{PCA}$ (AUC = 0.94), ${ {\mathcal H} }_{PCAL}$ and ${ {\mathcal H} }_{ALL}$ (AUC = 0.52). Among the individual modalities, ${ {\mathcal H} }_{ADAS}$, ${ {\mathcal H} }_{CSF}$ and ${ {\mathcal H} }_{APOE}$ provided the top 3 classification AUCs, which was consistent with observations in the training set which led to these three modalities being selected for fusion in CaMCCo.

For AD vs. MCI, ${ {\mathcal H} }_{CAMCCO}$, ${ {\mathcal H} }_{PCA}$ and ${ {\mathcal H} }_{ADAS}$ showed similar performances (AUC = 0.89). This may, in part, be on account of the lack of orthogonality in the features being fused. A correlation test between the ADAS and PET features showed correlation coefficients between −0.49 and −0.51 with p-value < 0.01. In fact, the modality selection strategy employed in this work is limited in that it does not account for relationships between modalities to identify those that optimize the performance when fused. Instead, the selection of modalities are simplified and are based on their individual performances. However, it is interesting to note that despite the significantly poorer performance of ${ {\mathcal H} }_{PET}$ as compared to ${ {\mathcal H} }_{ADAS}$, combining PET with ADAS-Cog does not degrade the performance. We note that, similar to HC vs. All, ${ {\mathcal H} }_{CAMCCO}$ significantly outperforms ${ {\mathcal H} }_{ALL}$.

### Experiment 2: Comparison of Multi-Class Classification Strategies for Fused Predictors

Table 6 shows the performance of ${ {\mathcal H} }_{CAMCCO}$, ${ {\mathcal H} }_{OVA}$ and ${ {\mathcal H} }_{OSC}$ for prediction of HC, MCI and AD. Across all 3 classes, it is evident that ${ {\mathcal H} }_{OVA}$ and ${ {\mathcal H} }_{CAMCCO}$ outperform ${ {\mathcal H} }_{OSC}$. ${ {\mathcal H} }_{OVA}$ and ${ {\mathcal H} }_{CAMCCO}$ show comparable AUCs for AD classification, although ${ {\mathcal H} }_{OVA}$ shows incrementally higher accuracy, sensitivity and specificity as compared to ${ {\mathcal H} }_{CAMCCO}$. For MCI classification however, ${ {\mathcal H} }_{CAMCCO}$ significantly outperforms ${ {\mathcal H} }_{OVA}$ in terms of all metrics (OVA AUC = 0.78 vs. CaMCCo AUC = 0.88). This is on account of the lower classification performance of MCI vs. all which is a challenging task provided the heterogeneity of the ‘all’ category which consists of both AD and HC patients. Therefore, CaMCCo provides the most optimal performance overall, across all 3 classes.

**Table 6:** Performance of multiclass classification strategies – one shot classifier (OSC), one vs. all (OVA), cascaded classifier in CaMCCo – upon fusion of modalities chosen from training set for each classification task.

		ACC	BACC	AUC	SEN	SPEC	PPV
CN	${ {\mathcal H} }_{OSC}$	0.69	0.63	0.96	0.34	0.92	0.75
	${ {\mathcal H} }_{OVA}$	**0**.**89**	**0**.**77**	**0**.**97**	**0**.**59**	**0**.**96**	**0**.**77**
	${ {\mathcal H} }_{CAMCCO}$	**0**.**89**	**0**.**77**	**0**.**97**	**0**.**59**	**0**.**96**	**0**.**77**
MCI	${ {\mathcal H} }_{OSC}$	0.69	0.69	0.77	0.68	**0**.**70**	0.67
	${ {\mathcal H} }_{OVA}$	0.68	0.68	0.77	0.78	0.59	0.63
	${ {\mathcal H} }_{CAMCCO}$	**0**.**80**	**0**.**78**	**0**.**89**	**0**.**88**	0.69	**0**.**80**
AD	${ {\mathcal H} }_{OSC}$	0.69	0.67	0.84	0.53	0.82	0.69
	${ {\mathcal H} }_{OVA}$	**0**.**85**	**0**.**82**	**0**.**90**	**0**.**72**	**0**.**91**	**0**.**81**
	${ {\mathcal H} }_{CAMCCO}$	0.80	0.78	0.89	0.69	0.88	0.80

The highest accuracy (ACC), balanced accuracy (BACC), area under the ROC curve (AUC), sensitivity (SEN), specificity (SPE) and positive predictive value (PPV) achieved for each class are shown in bold. These results indicate that although the performance of both ${ {\mathcal H} }_{OVA}$ and ${ {\mathcal H} }_{CAMCCO}$ are comparable for CN and AD classification, ${ {\mathcal H} }_{CAMCCO}$ outperforms all other methods for MCI classification.

### Experiment 3: Evaluation of Fused Representation on Binary Classification Tasks

Table 7 shows ${ {\mathcal H} }_{BIN}$ results obtained by combining select few modalities (${{\mathscr{K}}}_{SEL}$) via sMVCCA (${ {\mathcal F} }_{SMVCCA}$) for the following binary classification tasks: (i) AD vs. HC and (ii) MCI vs. HC. On the training set, the fusion of ADAS-Cog and CSF provided the best classification AUC for AD vs. HC whereas the fusion of ADAS-Cog, CSF and PET provided the best classification for MCI vs. HC. Thus, ${ {\mathcal H} }_{BIN}$ for the two classification tasks fused the respective, aforementioned modalities for the test set. For the former classification task, the performance of ${ {\mathcal H} }_{BIN}$ was similar to that of the best performing individual modality, ADAS-Cog, which already provided near perfect AUC leaving little scope for improvement. According to other evaluation metrics, ADAS-Cog outperforms the fused representation. For the more challenging MCI vs. HC classification task however, ${ {\mathcal H} }_{BIN}$ improves classifier performance slightly in terms of AUC (0.92 vs. 0.93) but more significantly in terms of BACC (0.77 vs. 0.82) and SPEC (0.65 vs. 0.71). In comparison to most previous work, our individual modality and fused modality results appear to be slightly higher possibly on account of the features that were considered in this work, all of which were quality controlled and independently proven to provide good performance previously. In addition, this work considers a neurocognitive score (ADAS-Cog), a measure that is mostly either used as a response variable or unconsidered in many fusion studies. The ADAS score appears to be strongly predictive of all classification tasks, possibly on account of a strong correlation with the MMSE scores, which were used to derive the ground truth class labels. Therefore, most gains in classification accuracy appears to be only slightly incremental. A correlation test between ADAS scores and MMSE scores across 118 patients, who had the latter data available, showed that the two were indeed highly correlated with a coefficient of −0.65 and p-value < 0.01.

**Table 7:** Performance of the combined fusion (${ {\mathcal F} }_{SMVCCA}$) and modality selection (${{\mathscr{K}}}_{SEL}$) modules of CaMCCo for binary classification (${ {\mathcal H} }_{BIN}$).

		ACC	BACC	AUC	SEN	SPEC	PPV
AD vs. HC	ADAS - Cog	**0**.**93**	**0**.**92**	**0**.**98**	**0**.**97**	**0**.**88**	**0**.**93**
	T1w MRI	0.63	0.55	0.65	0.86	0.24	0.66
	PET	0.80	0.78	0.87	0.86	0.71	0.83
	CSF	0.91	0.89	0.97	0.97	0.82	0.90
	PP	0.59	0.47	0.51	0.93	0.00	0.61
	APOE	0.76	0.70	0.86	0.93	0.47	0.75
	${ {\mathcal H} }_{BIN}$	0.87	0.85	**0**.**98**	0.93	0.76	0.87
MCI vs. HC	ADAS-Cog	0.79	0.75	0.84	0.85	0.65	0.85
	T1w MRI	0.69	0.49	0.53	0.98	0.00	0.70
	PET	0.72	0.67	0.76	0.80	0.53	0.80
	CSF	0.83	0.77	0.92	0.90	0.65	0.86
	PP	0.71	0.52	0.54	0.98	0.06	0.71
	APOE	0.83	0.71	0.84	**1**.**00**	0.41	0.80
	${ {\mathcal H} }_{BIN}$	**0**.**86**	**0**.**82**	**0**.**93**	0.93	**0**.**71**	**0**.**88**

${ {\mathcal H} }_{BIN}$ shows improvement over individual modality classifiers for MCI vs. HC, particularly in terms of achieving the both high sensitivity and specificity. For AD vs. HC, several individual modalities have sufficiently high classification performance and thereby leaving no room for further improvement with ${ {\mathcal H} }_{BIN}$.

## Conclusion

In this work, we present a joint cascaded classification and radio-omics data fusion framework, called Cascaded Multiview Canonical Correlation (CaMCCo), for early diagnosis of Alzheimer’s disease. CaMCCo employs a unique strategy as compared to most previous approaches in that it accounts for multiclass classification while attempting to optimize classification accuracy by fusing a select subset of modalities for prediction of each class. As a framework, CaMCCo is comprised of three modules: (i) data fusion, (ii) modality selection and (iii) multiclass classification. Experiments were designed to investigate the choice of methods used for each CaMCCo module independently. In the first experiment, for instance, classification method was held constant while the data fusion and modality selection modules were varied and compared with that of CaMCCo. In the second experiment, the modality selection and data fusion methods were held constant and the cascaded classifier in CaMCCo was compared against other multiclass classification methods. Experimental findings on the ADNI dataset, comprising imaging, proteomics, genomics and neurophysiological data, consistently indicated that fusion of select multi-scale data channels, as in CaMCCo, outperforms fusion of all available modalities. In addition, the results showed that cascaded classification used in CaMCCo is better suited than other multi-class classification methods for MCI prediction. Finally, CaMCCo was compared against individual modalities for the two most commonly investigated binary classification tasks in most related studies, AD vs. HC and MCI vs. HC. While AD vs. HC was a simpler task well resolved by a single modality in our study, MCI vs. HC was a more challenging task where the application of CaMCCo appeared to improve classification, most significantly in terms of specificity. CaMCCo appears to be better able to distinguish between MCI and HC as compared to most previous studies, some of which are listed in Table 5.

However, the work presented in this paper is limited mainly by the method with which the modalities to be combined at each level of the cascade is determined. We only combine the modalities that independently provide the best accuracies on the training set, which may not be complementary. Nonetheless, we found that considering a subset of modalities provides improved performance over fusing all modalities. These findings indicate that incorporation of a more advanced modality selection method and additionally a feature selection method into the framework may provide further improvement in performance. Another limitation of the proposed strategy is the propagation of error from one level of the cascade to the next. To minimize this error, we therefore begin the cascade with the one-vs-all classification providing the least error. Despite these limitations, current findings indicate that the presented framework provides a promising platform for fusion of multiscale, multimodal data for early diagnosis of Alzheimer’s Disease.

## Electronic supplementary material

Supplementary Information
