# Supplementary material for: Cascaded Multi-view Canonical Correlation (CaMCCo) for Early Diagnosis of Alzheimer’s Disease via Fusion of Clinical, Imaging and Omic Features
Source: Sci Rep. 2017 Aug 15;7:8137. doi: 10.1038/s41598-017-03925-0 (PMC5558022; doi:10.1038/s41598-017-03925-0)
Supplement: Supplementary file 1 — Supplementary Information [file 41598_2017_3925_MOESM1_ESM.pdf]

Cascaded Multi-view Canonical Correlation (CaMCCo) for Early Diagnosis of Alzheimer's Disease via Fusion of Clinical, Imaging and Omic Features

Asha Singanamalli, Haibo Wang, Alzheimer's Disease Neuroimaging Initiative, Anant Madabhushi

Appendix

RIDs of cases considered in this study

Training set: 10, 23, 33, 51, 101, 219, 221, 222, 231, 266, 285, 294, 319, 326, 341, 344, 361, 374, 394, 424, 470, 511, 535, 544, 547, 567, 577, 610, 627, 634, 657, 673, 680, 690, 720, 746, 783, 836, 866, 904, 925, 930, 932, 950, 1002, 1010, 1034, 1044, 1090, 1109, 1161, 1171, 1200, 1203, 1290, 1351, 1373, 1379, 1380, 1402
Test Set: 3, 8, 14, 47, 57, 95, 96, 120, 135, 147, 149, 150, 204, 213, 214, 240, 258, 286, 291, 292, 293, 316, 321, 362, 378, 386, 400, 431, 438, 454, 459, 464, 474, 481, 484, 492, 498, 543, 552, 565, 566, 598, 608, 621, 626, 637, 648, 672, 686, 723, 748, 751, 754, 800, 843, 850, 861, 891, 906, 941, 961, 973, 978, 994, 997, 1037, 1041, 1059, 1062, 1073, 1077, 1120, 1130, 1144, 1217, 1221, 1224, 1260, 1263, 1265, 1281, 1285, 1341, 1354, 1371, 1393, 1394, 1414, 1419

Theory and Review of CCA and MVCCA

Canonical Correlation Analysis

Provided a dataset $x_k^{n\times m}$ with $n \in \{1,2,...,N\}$ samples and $k \in \{1,2,...,K\}$ modalities, each of which comprises $m \in \{1,2...,M_k\}$ features. Canonical correlation analysis (CCA) considers two sets of variables ($K = 2$), $x_1^{n\times M_1}$ and $x_2^{n\times M_2}$, and projects them onto basis vectors, $\mathbf{w}_1$ and $\mathbf{w}_2$, such that correlation between projections of variables onto these basis vectors are mutually maximized. Formally, this can be expressed as

$$\underset{\mathbf{w}_1,\mathbf{w}_2}{\arg\max} \frac{\mathbf{w}_1^T \mathbf{C}_{12}\mathbf{w}_2}{\sqrt{\mathbf{w}_1^T \mathbf{C}_{11}\mathbf{w}_1 \mathbf{w}_2^T \mathbf{C}_{22}\mathbf{w}_2}}, \tag{5}$$

where $\mathbf{C}_{12} \in \mathbb{R}^{M_1\times M_2}$, $\mathbf{C}_{11} \in \mathbb{R}^{M_1\times M_1}$, $\mathbf{C}_{22} \in \mathbb{R}^{M_2\times M_2}$ are covariance matrices of $\mathbf{x_1}$ and $\mathbf{x_2}$, $\mathbf{x_1}$ and $\mathbf{x_1}$, and $\mathbf{x_2}$ and $\mathbf{x_2}$, respectively.

Multi-View Canonical Correlation Analysis (MVCCA)

Multiview CCA (MVCCA) can be derived by extending the CCA formulation to account for more than two sets of variables ($K > 2$). Since the joint correlation of more than two variables does not formally exist, MVCCA maximizes the sum of correlations between each pair of modalities. Thus, MVCCA can be expressed as generic form of Equation 5.

$$\underset{\mathbf{w}_1,...\mathbf{w}_k...,\mathbf{w}_K}{\arg\max} \sum_{k\neq j}\sum \frac{\mathbf{w}_k^T \mathbf{C}_{kj}\mathbf{w}_j}{\sqrt{\mathbf{w}_k^T \mathbf{C}_{kk}\mathbf{w}_k \mathbf{w}_j^T \mathbf{C}_{jj}\mathbf{w}_j}}. \tag{6}$$

The scaling of $\mathbf{w}$ does not affect the arg max solution, allowing Equation 6 to be written as:

$$\begin{aligned} \underset{\mathbf{w}_1,...,\mathbf{w}_K}{\arg\max} \quad & \sum_{k\neq j}\sum \mathbf{w}_k^T \mathbf{C}_{kj}\mathbf{w}_j \\ s.t. \quad & \mathbf{w}_1^T \mathbf{C}_{11}\mathbf{w}_1 = 1, \ldots, \mathbf{w}_K^T \mathbf{C}_{KK}\mathbf{w}_K = 1. \end{aligned} \tag{7}$$

Previously, Equation 6 has been solved by sequentially considering correlations of each pair of variables[32]. However, such an approach is sub-optimal as it requires iterative optimization, which is inefficient and can be susceptible to the order in which pairs of variable sets are chosen. Here, we present an alternative pairwise MVCCA approach by expressing correlations of all modalities in a combined correlation matrix which can be solved using eigenvalue decomposition method.
Letting $\mathbf{w} = [\mathbf{w}_1^T\ \mathbf{w}_2^T ... \mathbf{w}_K^T]^T$, $\mathbf{w} \in \mathbb{R}^{M\times 1}$ allows us to rewrite Equation 3 in a compact matrix form:

$$\begin{aligned} \underset{\mathbf{w}}{\arg\max} \quad & \mathbf{w}^T \bar{\mathbf{C}}\mathbf{w} \\ s.t \quad & \mathbf{w}^T \bar{\mathbf{C}}_d \mathbf{w} = 1 \\ & \mathbf{w}_1^T \mathbf{C}_{11}\mathbf{w}_1 = \ldots = \mathbf{w}_K^T \mathbf{C}_{KK}\mathbf{w}_K, \end{aligned} \tag{8}$$

where

$$\bar{\mathbf{C}} = \begin{bmatrix} \mathbf{0} & \mathbf{C}_{12} & \cdots & \mathbf{C}_{1K} \\ \mathbf{C}_{21} & \mathbf{0} & \ddots & \vdots \\ \vdots & \ddots & \ddots & \mathbf{C}_{(K-1)K} \\ \mathbf{C}_{K1} & \cdots & \mathbf{C}_{K(K-1)} & \mathbf{0} \end{bmatrix},$$

$$\bar{\mathbf{C}}_d = \begin{bmatrix} \mathbf{C}_{11} & \mathbf{0} & \cdots & \mathbf{0} \\ \mathbf{0} & \mathbf{C}_{22} & \ddots & \vdots \\ \vdots & \ddots & \ddots & \mathbf{0} \\ \mathbf{0} & \cdots & \mathbf{0} & \mathbf{C}_{KK} \end{bmatrix}. \tag{9}$$

In more general terms where $\mathbf{W} \in \mathbb{R}^{M\times n}$, Equation 4 reduces to

$$\begin{aligned} \arg\max_{\mathbf{W}} \quad & trace(\mathbf{W}_x^T \bar{\mathbf{C}} \mathbf{W}_x) \\ s.t \quad & \mathbf{W}_x^T \bar{\mathbf{C}}_d \mathbf{W}_x = \mathbf{I} \\ & \mathbf{w}_1^T \mathbf{C}_{11} \mathbf{w}_1 = \ldots = \mathbf{w}_K^T \mathbf{C}_{KK} \mathbf{w}_K, \end{aligned}$$

where $\mathbf{I}$ is an $n \times n$ identity matrix and the weight matrix is defined as $\mathbf{W}_x = [\mathbf{W}_1, \mathbf{W}_2, \ldots, \mathbf{W}_K] \in \mathbb{R}^{M\times n}$

Evaluation Metrics

**Metric**	**Definition**	**Description**
Accuracy (ACC)	$\frac{TP+TN}{TP+FP+TN+FN}$	Fraction of correctly identified positives and negatives
Sensitivity (SEN)	$\frac{TP}{TP+FN}$	Fraction of correctly identified positives
Specificity (SPE)	$\frac{TN}{TN+FP}$	Fraction of correctly identified negatives
Positive Predictive Value (PPV)	$\frac{TP}{TP+FP}$	Accuracy within samples identified as positive
Balanced Accuracy (BACC)[42]	$\frac{SEN+SPE}{2}$	Average accuracy of each class
Area Under the Curve (AUC)[43]	$\int_0^1 SENd(1-SPE)$	Area under the ROC Curve

Table 8. Description of all the evaluation metrics reported in this study; Abbreviations: True Positives (TP), False Positives (FP), True Negatives (TN), False Negatives (FN)
